# Dental Rehabilitation Improves Oral Health-Related Quality of Life in Children with Molar-Incisor Hypomineralisation: A 12-Month Prospective Controlled Study

**DOI:** 10.3390/children13050702

**Published:** 2026-05-20

**Authors:** Elif Kandemir Ülker, Seçil Çalışkan

**Affiliations:** Department of Pediatric Dentistry, Faculty of Dentistry, Eskişehir Osmangazi University, Eskişehir 26040, Türkiye; 523120211003@ogrenci.ogu.edu.tr

**Keywords:** molar incisor hypomineralisation (MIH), oral health-related quality of life (OHRQoL), pediatric dentistry, pediatric oral health-related quality of life (POQL)

## Abstract

**Highlights:**

**What are the main findings?**

• Children with MIH showed significantly poorer oral health–related quality of life (OHRQoL) compared with caries-matched controls.
• Following comprehensive dental rehabilitation, OHRQoL scores improved significantly in both groups, and children with MIH reached levels comparable to controls at 12-month follow-up.

**What are the implications of the main findings?**

• MIH negatively affects children’s quality of life independently of caries experience, highlighting the need for early diagnosis and targeted management.
• Comprehensive dental rehabilitation provides sustained improvements in OHRQoL, supporting its role as an effective long-term treatment strategy in children with MIH.

**Abstract:**

**Background/Objectives**: This study aims to compare the oral health-related quality of life (OHRQoL) of children with molar incisor hypomineralisation (MIH) and their parents with that of an age- and caries-matched control group, and to evaluate the long-term impact of dental rehabilitation on OHRQoL. **Methods**: A total of 30 children aged 8–12 years were included, with 15 participants in the MIH group and 15 in the control group. OHRQoL was assessed using the Pediatric Oral Health-Related Quality of Life (POQL) scale. Data were obtained at baseline and at 3, 6, and 12 months after treatment. Statistical analyses included a chi-square test, independent samples *t*-test, Mann–Whitney U test and Friedman test, according to data distribution. **Results**: Baseline POQL scores were significantly higher in the MIH group compared with the control group for both children and parents (*p* = 0.020 and *p* = 0.036, respectively). Among subscales, emotional functioning scores in children and role and physical functioning scores in parents were significantly higher in the MIH group (*p* = 0.027 and *p* = 0.032, respectively). Following dental rehabilitation, POQL scores significantly decreased in both groups (*p* < 0.001), and this improvement was maintained throughout the 12-month follow-up period. **Conclusions**: MIH has a negative impact on the OHRQoL of both children and parents, regardless of caries experience. Comprehensive dental rehabilitation results in significant and sustained improvements in OHRQoL, indicating the importance of early and comprehensive management in children with MIH.

## 1. Introduction

Molar incisor hypomineralisation (MIH) is a qualitative enamel defect involving at least one permanent first molar and presents clinically as demarcated opacities [1,2,3]. The prevalence of MIH reported in the literature varies widely worldwide, ranging from 0.48% to 46.6%, and it is considered one of the most common developmental enamel defects in children [4,5]. Although its etiology remains unclear, MIH has been associated with various prenatal, perinatal, and postnatal factors [6,7,8,9].

Enamel affected by MIH has been shown to exhibit higher organic content and lower mineral density compared with sound enamel, resulting in a more porous and mechanically compromised structure. These structural alterations increase susceptibility to functional stress and predispose affected teeth to post-eruptive enamel breakdown [10,11]. Consequently, MIH is associated with various clinical problems such as hypersensitivity, increased caries risk, post-eruptive enamel breakdown, adhesive failure in restorative treatments, and increased need for dental treatment [12,13,14]. In cases where incisors are involved, esthetic concerns may also arise [15].

Hypersensitivity associated with MIH may result in severe pain in response to mild stimuli, such as thermal changes, tooth brushing, or even airflow. This may lead children to avoid oral hygiene practices, creating a vicious cycle of increased caries risk and worsening MIH-related symptoms [16,17]. As a result, children with MIH are at increased risk of repeated dental interventions compared with their healthy counterparts [18].

Clinical findings do not always fully reflect the impact of oral conditions on an individual’s psychosocial well-being. Therefore, OHRQoL has emerged as an important measure of the impact of an individual’s oral health on daily life, social interaction, and psychological status [15,19].

The European Academy of Paediatric Dentistry (EAPD) has highlighted the potential psychosocial impact of MIH on children’s quality of life in its 2022 clinical guideline [20]. MIH may negatively affect daily activities and contribute to social and psychological difficulties due to pain, hypersensitivity, and esthetic concerns [21,22,23]. In particular, involvement of incisors may adversely influence self-perception and social behavior, with children reportedly avoiding smiling [15]. However, existing evidence remains inconsistent. While some studies report no significant association between MIH and OHRQoL, others demonstrate a clear negative impact [15,21,24]. Moreover, studies evaluating the effect of dental treatment on OHRQoL in children with MIH are limited, and prospective studies assessing long-term outcomes are particularly scarce.

Therefore, the aim of this study was to compare the OHRQoL of children with MIH and their parents with that of an age- and caries-matched control group, and to evaluate the long-term effects of comprehensive dental rehabilitation.

The null hypotheses were as follows:

**H_0_1:**  *There would be no significant difference in OHRQoL between the MIH and control groups.*

**H_0_2:**  *There would be no significant change in OHRQoL following dental rehabilitation in either group.*

## 2. Materials and Methods

### 2.1. Study Design and Ethical Approval

**Figure 1:**
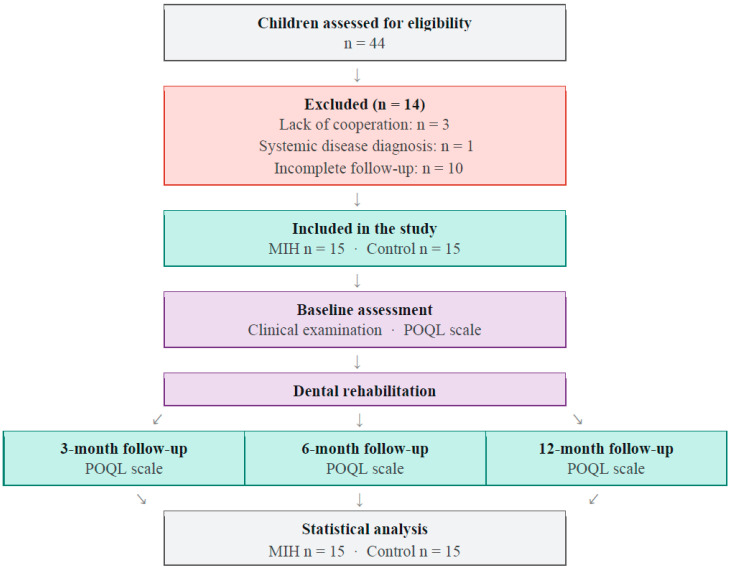
STROBE flow diagram of the study procedure.

This study was designed as a prospective controlled clinical study with a matched control group. Ethical approval was obtained from the Eskişehir Osmangazi University Non-Interventional Clinical Research Ethics Committee (decision dated 19 March 2024, no. 58). All participants were enrolled after written informed consent from their parents had been obtained. The study followed the STROBE guidelines recommended for observational research. The STROBE flow diagram illustrating the study procedure is presented in Figure 1 and was generated with the assistance of an artificial intelligence tool (Claude, Sonnet 4.6, Anthropic, San Francisco, CA, USA).

### 2.2. Participants and Group Allocation

Participants were selected from children aged 8–12 years who attended the Department of Pediatric Dentistry, Faculty of Dentistry, Eskişehir Osmangazi University, between May 2024 and February 2025. All clinical examinations and follow-up visits were performed by a single dentist (E.K.Ü.) with 5 years of clinical experience in pediatric dentistry.

The inclusion criteria were: systemically healthy children aged 8–12 years, in the mixed dentition period, with cooperative behavior (Frankl 3–4), and no prior restorative dental treatment. Participants not meeting these criteria were excluded.

The study group consisted of 15 children diagnosed with MIH. The control group included 15 children without MIH who met the same inclusion criteria and were matched to the MIH group in terms of age, gender, and caries experience (dmft/DMFT indices).

Participants who did not complete the treatment or follow-up period were excluded from the final analysis.

### 2.3. Sample Size Calculation

Sample size estimation was performed using G*Power software (version 3.1; Heinrich-Heine University Düsseldorf, Germany). Based on previous studies evaluating OHRQoL in children with MIH, a medium effect size (f = 0.40) was assumed. With a power of 80% and a significance level of 0.05, the minimum required sample size was calculated as 28 participants (14 per group) [25]. Considering potential dropouts during follow-up, a total of 30 participants (15 per group) were included.

### 2.4. Clinical Examination

A trained and calibrated examiner (E.K.Ü.) established the diagnosis of MIH based on EAPD criteria. The severity of MIH was determined in accordance with EAPD recommendations, and participants were classified as having mild or severe MIH based on the most severely affected tooth, as commonly used in the literature [17,20,21,26,27]. Dental caries assessment followed World Health Organization (WHO) criteria, and the dmft index for primary teeth and the DMFT index for permanent teeth were used to record caries experience. Intra-examiner reliability was assessed by re-evaluating a subset of participants after a two-week interval. Agreement was calculated using the kappa coefficient (κ = 0.86).

### 2.5. Treatment Protocol

All dental treatment procedures were performed by a pediatric dentist (E.K.Ü.) following standardized clinical protocols and the MIH Treatment Need Index [28]. Treatment plans and procedures were reviewed by a senior pediatric dentistry specialist (S.Ç., 15 years of experience) to ensure consistency.

**Figure 2:**
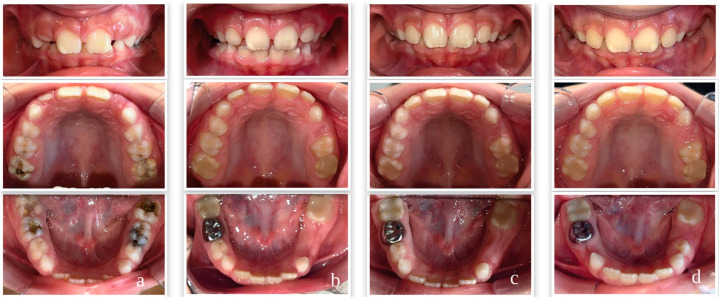
Case 1 – Intraoral photographs of a participant from the MIH group at baseline and at 3, 6, and 12 months post-treatment. (**a**) Baseline; (**b**) 3 months post-treatment; (**c**) 6 months post-treatment; (**d**) 12 months post-treatment.

**Figure 3:**
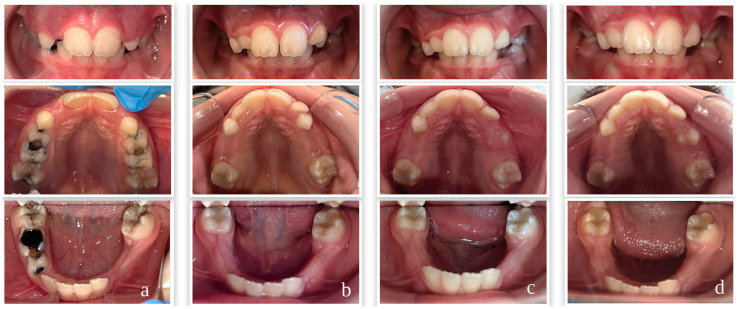
Case 2—Intraoral photographs of a participant from the MIH group at baseline and at 3, 6, and 12 months post-treatment. (**a**) Baseline; (**b**) 3 months post-treatment; (**c**) 6 months post-treatment; (**d**) 12 months post-treatment.

Comprehensive dental rehabilitation was provided to participants based on their individual treatment needs and included preventive procedures, restorative treatments, stainless steel crowns, vital pulp therapies, root canal treatments, and extractions when indicated, in accordance with current clinical guidelines [20,29,30,31,32,33,34]. Restorative treatments included composite resin for permanent teeth (Neo Spectra ST HV, Dentsply Sirona, Bensheim, Germany) and compomer for primary teeth (Dyract XP, Dentsply Sirona, Germany). Stainless steel crown applications utilized Kids Crown (Shinhung, Seoul, Republic of Korea), vital pulp therapies were performed using a bioactive bioceramic pulp treatment material (NeoPUTTY, NuSmile, Houston, TX, USA), and root canal treatment procedures employed bioceramic-based root canal sealer for permanent teeth (Ceraseal, Meta Biomed, Cheongju, Republic of Korea) and calcium hydroxide paste with iodoform for primary teeth (DiaPex Plus, DiaDent, Osong, Republic of Korea). All treatment procedures were completed within four sessions scheduled at one-week intervals. Representative intraoral photographs of two cases at baseline and post-treatment follow-up visits are presented in Figure 2 and Figure 3.

### 2.6. Outcome Measure: OHRQoL

OHRQoL was assessed using the POQL scale [19,35]. The scale was administered as both child self-report and parent proxy-report, in separate settings to prevent interaction between respondents. Children and parents completed the questionnaires independently with standardized clarification provided only when necessary. No leading or suggestive guidance was provided during administration. The Turkish version of POQL includes three dimensions: role and physical, social and emotional functioning. Scores were calculated according to the standard scoring protocol.

### 2.7. Data Collection and Follow-Up

The data collection was conducted in two phases. At baseline, demographic variables (age, gender), oral hygiene habits, caries experience (dmft/DMFT), parental education level, and income status were recorded. The POQL scale was administered to both children and parents, and oral hygiene instructions were provided. Following the completion of the dental treatment, participants were recalled at the 3rd, 6th, and 12th months. During the follow-up period, the POQL scale was re-administered. During the follow-up period, any additional treatment needs were managed according to standard clinical protocols, and a consistent approach was applied to all participants. Professional fluoride application was performed every three months.

### 2.8. Statistical Analysis

Statistical analyses were performed using IBM SPSS Statistics software (version 27.0; IBM Corp., Armonk, NY, USA). Descriptive statistics were calculated as mean ± standard deviation or median (min–max) for continuous variables and frequencies (*n*, %) for categorical variables.

Data distribution was evaluated with the Shapiro–Wilk test, and equality of variances was checked using Levene’s test. Depending on these results, between-group comparisons were carried out using either the independent samples *t*-test or the Mann–Whitney U test.

Within-group comparisons across time points (baseline, 3, 6, and 12 months) were analyzed using Friedman test. Post hoc analyses were conducted using Bonferroni correction or Wilcoxon signed-rank test. Categorical variables were analyzed using the chi-square test.

To assess the independent effect of group and time on OHRQoL while controlling for potential confounders, a linear mixed-effects model was employed separately for child- and parent-reported outcomes. Group (MIH vs. control), time (baseline, 3, 6, and 12 months), and their interaction (group × time) were entered as fixed effects. Income, parental education level, and oral hygiene habits were included as covariates. Participant ID was specified as a random effect to account for within-subject correlation, and a first-order autoregressive [AR(1)] covariance structure was applied to the repeated measurements.

A *p*-value of <0.05 was considered statistically significant. Although multiple statistical tests were applied, a global correction for multiple comparisons was not performed, as all comparisons were pre-specified based on the study hypotheses and the analyses were considered exploratory.

## 3. Results

### 3.1. Participant Flow and Baseline Characteristics

A total of 44 children (24 with MIH and 20 controls) were initially included in the study. During the study, 3 participants were excluded due to lack of cooperation, 1 due to the diagnosis of a systemic disease, and 10 due to incomplete follow-up. Therefore, the final analysis included 30 participants (15 per group).

The mean age of the participants was 8.59 ± 0.73 years, with no significant differences between the groups in terms of age and gender distribution (MIH: 8.47 ± 0.78; control: 8.70 ± 0.69; *p* = 0.455; 53.33% female, 46.67% male; *p* = 0.715). Similarly, no significant differences were observed between the MIH and control groups regarding caries experience (dmft: *p* = 0.896; DMFT: *p* = 0.404), oral hygiene habits, parental education level, or income status (*p* = 0.567; *p* = 0.660; *p* = 0.492, respectively).

The distribution and types of dental treatments were comparable between groups (restorative: *p* = 0.851; endodontic: *p* = 0.283; extraction: *p* = 0.863). Patients in the MIH group received a mean of 6.33 ± 1.68 restorative, 0.87 ± 0.91 endodontic, and 3.73 ± 1.87 extraction treatments, whereas control group patients received 6.20 ± 2.14 restorative, 0.53 ± 0.74 endodontic, and 3.87 ± 2.30 extraction treatments.

In the MIH group, incisor involvement was present in 53.3% of children, and hypersensitivity in 66.7%. Regarding MIH severity, 46.7% of cases were classified as mild and 53.3% as severe. Neither hypersensitivity nor severity was associated with a statistically significant difference in baseline POQL scores (*p* > 0.05).

### 3.2. Changes in Total POQL Scores

The distribution of total POQL scores across groups and time points is presented in Table 1.

At baseline, both child- and parent-reported POQL scores were significantly higher in the MIH group compared to the control group (*p* = 0.020 and *p* = 0.036, respectively). Following dental rehabilitation, POQL scores significantly decreased in both groups (*p* < 0.001). No statistically significant differences were observed between the groups at 3 months or at any subsequent follow-up time point for either child- or parent-reported scores (*p* > 0.05) (Table 1).

The linear mixed-effects model revealed a significant group × time interaction for both child-reported (F(3, 52.43) = 5.796, *p* = 0.002) and parent-reported POQL scores (F(3, 57.20) = 4.521, *p* = 0.006), indicating that the two groups followed different trajectories of change over time. The MIH group demonstrated substantially higher scores at baseline (child: 20.13; parent: 20.86) compared to the control group (child: 11.04; parent: 10.47), with both groups converging to similarly low scores by the 12-month follow-up. Importantly, none of the covariates—income, parental education, or oral hygiene—reached statistical significance (all *p* > 0.05), indicating that the observed group × time effect was not confounded by these socioeconomic or behavioral variables. These findings are presented in Table 2 and Table 3.

Child- and parent-reported POQL scores showed a strong positive correlation (r = 0.709, *p* < 0.001).

### 3.3. POQL Subscale Analysis

The distribution of POQL subscale scores according to group and time point is presented in Table 4.

### 3.4. Child-Reported Outcomes

Role/physical and social functioning scores did not differ significantly between the MIH and control groups at any time point (*p* > 0.05). Emotional functioning scores were significantly higher in the MIH group at baseline (*p* = 0.027); however, no differences were observed at 3-, 6-, or 12-month follow-ups (*p* > 0.05). Within-group analyses demonstrated a significant decrease in all subscale scores over time in both groups (MIH: *p* < 0.001 for all; control: *p* < 0.05 for all).

### 3.5. Parent-Reported Outcomes

Social and emotional functioning scores did not differ significantly between groups at any time point (*p* > 0.05). Role and physical functioning scores were significantly higher in the MIH group at baseline (*p* = 0.032), but no significant differences were observed at follow-up assessments (*p* > 0.05). A significant decrease in subscale scores was observed over time in the MIH group for all domains (*p* < 0.05). In the control group, significant decreases were observed in role/physical and emotional functioning (*p* < 0.001; *p* = 0.002, respectively), whereas no statistically significant change was detected in social functioning scores (*p* = 0.194).

### 3.6. Effect of Incisor Involvement

Among children with MIH, those with incisor involvement had significantly higher baseline social functioning scores compared to those without incisor involvement (*p* = 0.042). No significant differences were observed in parent-reported outcomes (*p* = 0.980).

### 3.7. General Health Perceptions and Treatment Impact

When responses to the general questions were analyzed, the most common reason for the first dental visit in both groups was emergency dental pain (MIH: 46.7%; control: 40.0%), followed by restorative treatment (MIH: 40.0%; control: 26.7%).

At baseline, a significantly higher proportion of children in the MIH group rated their dental health as poor compared to the control group (46.7% vs. 13.3%, *p* = 0.046). This proportion decreased significantly after treatment in the MIH group (*p* < 0.001). Similarly, although a higher proportion of parents in the MIH group rated their children’s dental health as poor at baseline, the difference was not statistically significant (53.3% vs. 20.0%; *p* = 0.058). However, a significant reduction was observed following treatment (*p* < 0.001).

When perceived changes in dental health compared to one year earlier were evaluated, no significant differences were observed between the groups at baseline for either children or parents (*p* > 0.05). At the 3-month follow-up, most participants in both groups reported improvement, and by the 6- and 12-month follow-ups, all children and parents in both groups reported better dental health compared to one year earlier. Within-group comparisons showed significant improvements over time in both groups (*p* < 0.001).

## 4. Discussion

MIH is considered an important public health issue due to its high global prevalence and the substantial treatment burden it imposes on affected children and their families. In this context, understanding its impact on OHRQoL and the extent to which this impact can be modified through dental interventions is of clinical and public health importance [36].

Although previous studies have demonstrated the negative impact of MIH on OHRQoL, most have employed cross-sectional designs or have been limited to short-term pre- and post-treatment evaluations [17,25,37,38,39]. Consequently, evidence regarding the sustainability of treatment-related improvements remains limited. The present study fills this gap by providing prospective data with a 12-month follow-up and by comparing outcomes with a control group matched for age, gender, and caries experience.

The selected age range reflects the period during which permanent first molars have erupted and untreated lesions are more likely to be identified, ensuring an appropriate clinical context for MIH assessment. The age distribution of the participants is consistent with previous studies on MIH and quality of life [15,17,21,37,39,40]. In addition, the use of the POQL scale, incorporating both child self-reports and parent proxy-reports, represents a key strength of the study. This approach enables a more comprehensive evaluation of OHRQoL and has been shown to sensitively capture the impact of dental conditions, particularly caries-related factors [19].

One of the major strengths of this study is the use of a control group matched for age, gender, and caries experience (dmft/DMFT). This design reduces the potential confounding effect of caries on OHRQoL and allows a more reliable interpretation of differences attributable to MIH. In contrast, previous studies have often included control groups with differing caries experience or caries-free individuals, which may limit the ability to distinguish whether observed differences in OHRQoL are related to MIH or underlying caries status [21,22,40].

In the present study, children with MIH exhibited significantly poorer OHRQoL at baseline compared to controls, as reported by both children and parents. These findings are consistent with previous studies reporting an association between MIH and impaired OHRQoL [15,17,39]. A study using the POQL scale reported higher OHRQoL scores in children with MIH compared to controls, although no significant differences were observed in parent-reported outcomes [17]. This discrepancy may be related to the lack of balance in caries experience between groups in that study. In particular, differences in DMFS scores may have limited the ability to attribute the observed effects solely to MIH. In contrast, the use of a caries-matched control group in the present study strengthens the interpretation that the observed differences are more likely attributable to MIH rather than underlying differences in caries experience.

The findings of this study demonstrate that full-mouth dental rehabilitation reduces the negative impact of MIH on OHRQoL, with post-treatment scores in the MIH group reaching levels comparable to those of the control group. Importantly, this improvement was sustained throughout the 12-month follow-up period. Previous studies have consistently reported improvements in OHRQoL following dental treatment in children with MIH [12,14,37,39]. Altner et al. also observed significant post-treatment improvements in both MIH and control groups [40]. Likewise, Bekes et al. demonstrated short-term improvements following hypersensitivity-focused treatments [25]. However, the available evidence predominantly reflects short-term outcomes, and data on the durability of these improvements remain limited. Within this framework, the present study extends the existing evidence by demonstrating that the beneficial effects of comprehensive dental rehabilitation can be maintained for at least 12 months.

Analysis of POQL subscales indicated that emotional functioning was the most affected domain at baseline in children with MIH. Although the MIH group demonstrated higher scores across all subscales compared to controls, a statistically significant difference was observed only in the emotional functioning domain. This dimension reflects emotional responses such as irritability, anxiety, sadness, and crying related to dental conditions. Previous studies have also shown that children with MIH are more likely to experience negative emotional responses associated with oral health problems [15,38,40]. Similarly, Kisacik et al. reported significantly higher emotional functioning scores in children with MIH compared to those without MIH [17]. Following treatment, significant improvements were observed across all subscales in both MIH and control groups, with the most notable changes seen in emotional functioning. This finding supports the multidimensional benefit of dental rehabilitation. In line with these results, Altner et al., using the Child Perception Questionnaire (CPQ), reported improvements in emotional and social well-being as well as functional limitation domains after dental treatment [40].

In terms of parent-reported outcomes, higher baseline role and physical functioning scores were observed in the MIH group compared to controls. This finding is in line with the study by Kisacik et al. [17] and may be explained by parents’ greater ability to recognize observable symptoms such as pain and difficulty in eating.

Responses to the general questions indicated that children with MIH and their parents were more likely to report dissatisfaction with oral health at baseline. While general question findings were not reported in the POQL-based study by Kisacik et al., Altner et al. reported similar findings using the CPQ, where a higher proportion of children with MIH rated their oral health as poor, and this perception improved following dental treatment [17,40].

This study has several limitations, including reliance on self-reported data and a relatively small sample size. Despite a one-year recruitment period, the difficulty in identifying children with MIH who had no prior treatment history and met the inclusion criteria, as well as the demands of long-term follow-up, limited the sample size. This may restrict the generalizability of the findings. A further limitation is that OHRQoL is a multifactorial construct, and since both groups received comprehensive dental treatment, the improvements observed may reflect the general effects of dental rehabilitation rather than being attributable to MIH management alone. Additionally, the single-center and single-operator design may limit the generalizability of the findings, and the absence of a dropout analysis for participants lost to follow-up may have introduced attrition bias. Further research with larger cohorts and longer follow-up is needed to support these findings. In addition, the broader psychosocial burden of MIH on families and its potential impact on children’s academic performance warrant further investigation.

Despite these limitations, the study has several strengths. These include the inclusion of untreated MIH cases, the use of a caries-matched control group, and the evaluation of treatment outcomes over a 12-month follow-up period. This design allows a more reliable assessment of the independent effect of MIH on OHRQoL and provides valuable insight into the sustainability of treatment outcomes.

## 5. Conclusions

The findings indicate that MIH affects multiple dimensions of OHRQoL, particularly emotional well-being and physical functioning. The comparison of groups with similar caries experience revealed that MIH has an independent negative effect on OHRQoL.

Following full-mouth dental rehabilitation, significant improvements in OHRQoL were observed in both the MIH and control groups. The scores of children with MIH reached control group levels and these improvements were sustained over a 12-month follow-up period.

These findings highlight not only the short-term benefits of treatment but also the sustainability of long-term well-being, supporting the clinical importance of early and comprehensive treatment approaches in children with MIH.

## Figures and Tables

**Table 1:** Total POQL scores in MIH and control groups across follow-up time points.

	MIH		Control		*p*
Child	Med	min–max	Med	min – max	
Baseline	20.80 ^a^	5.00–35.00	9.20 ^a^	0–25.80	0.020 *
3rd Month	0 ^b^	0–10.00	1.70 ^b^	0–12.50	0.528
6th Month	0 ^b^	0–5.00	0 ^b^	0–11.70	0.486
12th Month	0 ^b^	0–3.30	0 ^b^	0–3.30	0.614
*p*	<0.001 *		<0.001 *		
Parent	Med	min–max	Med	min–max	
Baseline	18.30 ^a^	2.50–49.20	7.50 ^a^	0–27.50	0.036 *
3rd Month	0 ^b^	0–23.30	0 ^ab^	0–13.30	0.851
6th Month	0 ^b^	0–4.20	0 ^b^	0–15.00	0.812
12th Month	0 ^b^	0–5.00	0 ^b^	0–5.00	0.577
*p*	<0.001 *		<0.001 *		

* Designates statistically significant difference. Different superscript letters indicate statistically significant differences among groups (*p* < 0.05).

**Table 2:** Linear mixed-effects model—Type III Tests of Fixed Effects.

	Child		Parent	
	F (df)	*p*	F (df)	*p*
Time	48.271 (3, 52.43)	<0.001 *	31.822 (3, 57.20)	<0.001 *
Group	2.317 (1, 21.83)	0.142	3.148 (1, 23.32)	0.089
Income	1.181 (1, 22.20)	0.289	0.052 (1, 23.31)	0.821
Parental Education	0.190 (1, 22.20)	0.667	0.492 (1, 23.31)	0.490
Oral Hygiene	0.071 (1, 22.20)	0.792	0.287 (1, 23.31)	0.597
Time × Group	5.796 (3, 52.43)	0.002 *	4.521 (3, 57.20)	0.006 *

* *p* < 0.05 statistically significant. df = degrees of freedom (numerator, denominator). Covariates (income, parental education, oral hygiene) were evaluated at their mean values: income = 1.30, parental education = 0.93, oral hygiene = 1.70.

**Table 3:** Estimated marginal means of POQL scores by group and time point, adjusted for income, parental education, and oral hygiene.

	Child		Parent	
	MIH	Control	MIH	Control
Baseline	20.13	11.04	20.86	10.47
3rd Month	2.31	2.75	4.03	2.91
6th Month	1.35	2.65	0.48	1.81
12th Month	0.75	0.37	0.25	0.53

Covariates were evaluated at their mean values: income = 1.30, parental education = 0.93, and oral hygiene = 1.70. Values represent adjusted means.

**Table 4:** POQL subscale scores in MIH and control groups across follow-up.

	Child Scores					Parent Scores				
	MIH		Control		*p*	MIH		Control		*p*
Role and Physical Functioning	Med	min–max	Med	min–max		Med	min–max	Med	min – max	
Baseline	9.20 ^a^	1.70–2.80	6.70 ^a^	0–20.00	0.134	10.00 ^a^	0–20.00	5.00 ^a^	0–12.50	0.032 *
3rd Month	0 ^b^	0–10.00	0 ^b^	0–8.30	0.939	0 ^b^	0–6.70	0 ^b^	0–6.70	0.570
6th Month	0 ^b^	0–5.00	0 ^b^	0–8.30	0.337	0 ^b^	0–2.50	0 ^b^	0–6.60	0.962
12th Month	0 ^b^	0–3.30	0 ^b^	0–3.30	0.953	0 ^b^	0–5.00	0 ^b^	0–5.00	0.577
*p*	<0.001 *		<0.001 *			<0.001 *		<0.001 *		
Social Functioning	Med	min–max	Med	min–max		Med	min–max	Med	min – max	
Baseline	2.50 ^a^	0–10.00	0 ^a^	0–5.00	0.452	0 ^a^	0–9.20	0 ^a^	0–20.00	0.328
3rd Month	0 ^b^	0–1.70	0 ^b^	0–0	0.317	0 ^ab^	0–9.20	0 ^a^	0–0	0.073
6th Month	0 ^b^	0–3.30	0 ^b^	0–0	0.150	0 ^ab^	0–4.20	0 ^a^	0–6.70	0.550
12th Month	0 ^b^	0–0	0 ^b^	0–0		0 ^b^	0–0	0 ^a^	0–0	
*p*	<0.001 *		<0.001 *			0.031 *		0.194		
Emotional Functioning	Med	min–max	Med	min–max		Med	min–max	Med	min–max	
Baseline	5.00 ^a^	0–25.00	1.70 ^a^	0–12.50	0.027 *	5.80 ^a^	0–21.70	2.50 ^a^	0–15.00	0.314
3rd Month	0 ^b^	0–10.00	0 ^ab^	0–10.00	0.061	0 ^ab^	0–15.00	0 ^ab^	0–8.30	0.558
6th Month	0 ^b^	0–2.50	0 ^ab^	0–10.00	0.498	0 ^ab^	0–1.70	0 ^b^	0–8.30	0.498
12th Month	0 ^b^	0–2.50	0 ^b^	0–0	0.317	0 ^b^	0–0	0 ^b^	0–0	
*p*	<0.001 *		0.005 *			<0.001 *		0.002 *		

* Designates statistically significant difference. Different superscript letters indicate statistically significant differences among groups (*p* < 0.05).

## Data Availability

The data supporting the findings of this study are available from the corresponding author upon reasonable request.

## Abbreviations

The following abbreviations are used in this manuscript:

OHRQoL	Oral Health-Related Quality of Life
MIH	Molar Incisor Hypomineralisation
POQL	Pediatric Oral Health-Related Quality of Life
STROBE	Strengthening The Reporting of Observational Studies in Epidemiology
EAPD	The European Academy of Paediatric Dentistry
WHO	World Health Organization
CPQ	Child Perception Questionnaire

## References

[1] Mastroberardino S., Campus G., Strohmenger L., Villa A., Cagetti M.G. (2012). An Innovative Approach to Treat Incisors Hypomineralization (MIH): A Combined Use of Casein Phosphopeptide-Amorphous Calcium Phosphate and Hydrogen Peroxide-A Case Report. Case Rep. Dent..

[2] Weerheijm K.L., Duggal M., Mejàre I., Papagiannoulis L., Koch G., Martens L.C., Hallonsten A.L. (2003). Judgement criteria for molar incisor hypomineralisation (MIH) in epidemiologic studies: A summary of the European meeting on MIH held in Athens, 2003. Eur. J. Paediatr. Dent..

[3] Weerheijm K.L. (2003). Molar incisor hypomineralisation (MIH). Eur. J. Paediatr. Dent..

[4] Sluka B., Held U., Wegehaupt F., Neuhaus K.W., Attin T., Sahrmann P. (2024). Is there a rise of prevalence for Molar Incisor Hypomineralization? A meta-analysis of published data. BMC Oral Health.

[5] Mast P., Rodrigueztapia M.T., Daeniker L., Krejci I. (2013). Understanding MIH: Definition, epidemiology, differential diagnosis and new treatment guidelines. Eur. J. Paediatr. Dent..

[6] Bandeira Lopes L., Machado V., Botelho J., Haubek D. (2021). Molar-incisor hypomineralization: An umbrella review. Acta Odontol. Scand..

[7] Alhowaish L., Baidas L., Aldhubaiban M., Bello L.L., Al-Hammad N. (2021). Etiology of Molar-Incisor Hypomineralization (MIH): A Cross-Sectional Study of Saudi Children. Children.

[8] Silva M.J., Scurrah K.J., Craig J.M., Manton D.J., Kilpatrick N. (2016). Etiology of molar incisor hypomineralization—A systematic review. Community Dent. Oral Epidemiol..

[9] Butera A., Maiorani C., Morandini A., Simonini M., Morittu S., Barbieri S., Bruni A., Sinesi A., Ricci M., Trombini J. (2021). Assessment of genetical, pre, peri and post natal risk factors of deciduous molar hypomineralization (DMH), hypomineralized second primary molar (HSPM) and molar incisor hypomineralization (MIH): A narrative review. Children.

[10] Suckling G.W. (1989). Developmental defects of enamel—historical and present-day perspectives of their pathogenesis. Adv. Dent. Res..

[11] Elhennawy K., Schwendicke F. (2016). Managing molar-incisor hypomineralization: A systematic review. J. Dent..

[12] Tugcu N., Sezer B., Caliskan C., Altinok Durmus B., Kargul B. (2022). Changes in oral health-related quality of life after treatment of molar incisor hypomineralization using Glass Hybrid Restorations. J. Pak. Med. Assoc..

[13] Oyedele T.A., Folayan M.O., Adekoya-Sofowora C.A., Oziegbe E.O. (2015). Co-morbidities associated with molar-incisor hypomineralisation in 8 to 16 year old pupils in Ile-Ife, Nigeria. BMC Oral Health.

[14] Sekundo C., Jung M., Muscholl C., Frese C. (2024). Oral health-related quality of life and survival analysis after preventive and restorative treatment of molar-incisor hypomineralisation. Sci. Rep..

[15] Joshi T., Rahman A., Rienhoff S., Rienhoff J., Stamm T., Bekes K. (2022). Impact of molar incisor hypomineralization on oral health-related quality of life in 8–10-year-old children. Clin. Oral Investig..

[16] Weerheijm K.L. (2004). Molar incisor hypomineralization (MIH): Clinical presentation, aetiology and management. Dent. Update.

[17] Kisacik S., Ozler C.O., Olmez S. (2024). Molar incisor hypomineralization and oral health-related quality of life: A sample of 8–12-years-old children. Clin. Oral Investig..

[18] Kotsanos N., Kaklamanos E.G., Arapostathis K. (2005). Treatment management of first permanent molars in children with Molar-Incisor Hypomineralisation. Eur. J. Paediatr. Dent..

[19] Yazıcıoğlu İ., Jones J., Doğan C., Rich S., Garcia R.I. (2018). Validity and reliability of a Turkish pediatric oral health-related quality of life measure. Eur. Oral Res..

[20] Lygidakis N.A., Garot E., Somani C., Taylor G.D., Rouas P., Wong F.S.L. (2022). Best clinical practice guidance for clinicians dealing with children presenting with molar-incisor-hypomineralisation (MIH): An updated European Academy of Paediatric Dentistry policy document. Eur. Arch. Paediatr. Dent..

[21] Elhennawy K., Rajjoub O., Reissmann D., Doueiri M.-S., Hamad R., Sierwald I., Wiedemann V., Bekes K., Jost-Brinkmann P.-G. (2022). The association between molar incisor hypomineralization and oral health-related quality of life: A cross-sectional study. Clin. Oral Investig..

[22] Michaelis L., Ebel M., Bekes K., Klode C., Hirsch C. (2021). Influence of caries and molar incisor hypomineralization on oral health-related quality of life in children. Clin. Oral Investig..

[23] Hoogeveen R.P.P., Momayez E., Bonifacio C.C., Manton D.J., Hesse D. (2025). Impact of treatment of molar-incisor hypomineralisation on children’s oral health-related quality of life: A systematic review. Eur. Arch. Paediatr. Dent..

[24] Awwad A., Hamad R., Schiffner U., Splieth C., Schmoeckel J. (2023). Effect of prevalence and severity of molar-incisor hypomineralization on oral health-related quality of life: A systematic review and meta-analysis. Acta Stomatol. Croat..

[25] Bekes K., Amend S., Priller J., Zamek C., Stamm T., Krämer N. (2021). Changes in oral health-related quality of life after treatment of hypersensitive molar incisor hypomineralization-affected molars with a sealing. Clin. Oral Investig..

[26] Marcianes M., García-Camba P., Albaladejo A., Varela Morales M. (2023). Predictive Value of Hypomineralization of Second Primary Molars for Molar Incisor Hypomineralization and Other Relationships Between Both Developmental Defects of Dental Enamel. J. Clin. Med..

[27] Medina Varela A.F., García Pérez A., Villanueva Gutiérrez T., Mora Navarrete K.A., Nieto Sánchez M.P. (2024). An inverse relationship between dental fluorosis and Molar Incisor Hypomineralization in Mexican schoolchildren in an area with a high concentration of fluoride in drinking water: A cross-sectional study. PLoS ONE.

[28] Bekes K., Steffen R., Krämer N. (2023). Update of the molar incisor hypomineralization: Würzburg concept. Eur. Arch. Paediatr. Dent..

[29] Wright J.T., Crall J.J., Fontana M., Gillette E.J., Nový B.B., Dhar V., Donly K., Hewlett E.R., Quinonez R.B., Chaffin J. (2016). Evidence-based clinical practice guideline for the use of pit-and-fissure sealants: A report of the American Dental Association and the American Academy of Pediatric Dentistry. J. Am. Dent. Assoc..

[30] Coll J.A., Dhar V., Vargas K., Chen C.Y., Crystal Y.O., AlShamali S., Marghalani A.A. (2020). Use of Non-Vital Pulp Therapies in Primary Teeth. Pediatr. Dent..

[31] American Academy of Pediatric Dentistry (2025). Pediatric Restorative Dentistry. The Reference Manual of Pediatric Dentistry.

[32] Duggal M., Gizani S., Albadri S., Krämer N., Stratigaki E., Tong H.J., Seremidi K., Kloukos D., BaniHani A., Santamaría R.M. (2022). Best clinical practice guidance for treating deep carious lesions in primary teeth: An EAPD policy document. Eur. Arch. Paediatr. Dent..

[33] Coll J.A., Dhar V., Chen C.Y., Crystal Y.O., Guelmann M., Marghalani A.A., AlShamali S., Xu Z., Glickman G.N., Wedeward R. (2024). Use of Vital Pulp Therapies in Primary Teeth 2024. Pediatr. Dent..

[34] Coll J.A., Dhar V., Guelmann M., Crystal Y.O., Chen C.Y., Marghalani A.A., AlShamali S., Xu Z., Ather A., Sabeti M. (2025). Guideline for Use of Vital Pulp Therapy in Permanent Teeth. Pediatr. Dent..

[35] Huntington N.L., Spetter D., Jones J.A., Rich S.E., Garcia R.I., Spiro A. (2011). Development and validation of a measure of pediatric oral health-related quality of life: The POQL. J. Public Health Dent..

[36] Schneider P.M., Silva M. (2018). Endemic Molar Incisor Hypomineralization: A Pandemic Problem That Requires Monitoring by the Entire Health Care Community. Curr. Osteoporos. Rep..

[37] Jiménez-Lobo J., Batista-Cárdenas D., Aguilar-Cubillo A., Gómez-Fernández A., Ramírez K. (2023). Changes in oral health-related quality of life before and after dental treatment in 8–12-year-old Costa Rican schoolchildren. Front. Dent. Med..

[38] Gadallah L.K., Korayem E., Wahby R. (2024). Oral health-related quality of life in Egyptian children with Molar Incisor Hypomineralisation. An observational study. BDJ Open.

[39] Samha H., Walia T., Shetty R.M., Baroudi K., Hashim R., Berdouses E.D. (2025). Influence of molar incisor hypomineralization on oral health-related quality of life in 8–10-year-old children: A cross-sectional study. Eur. Arch. Paediatr. Dent..

[40] Altner S., Ebel M., Ritschl V., Stamm T., Hirsch C., Bekes K. (2022). Treatment of severe caries and molar incisor hypomineralization and its influence on oral health-related quality of life in children: A comparative study. Int. J. Environ. Res. Public Health.

